# Supplementary material for: Integrating Self-Report and Psychophysiological Measures in Waterpipe Tobacco Message Testing: A Novel Application of Multi-Attribute Decision Modeling
Source: Int J Environ Res Public Health. 2021 Nov 11;18(22):11814. doi: 10.3390/ijerph182211814 (PMC8617707; doi:10.3390/ijerph182211814)
Supplement: Supplementary file 1 [file ijerph-18-11814-s001.zip › ijerph-1395265-supplementary.pdf]

**Table S1.** Message Examples.

| Message Identifier | Message |
| --- | --- |
| H1:<br>Harms 1 | 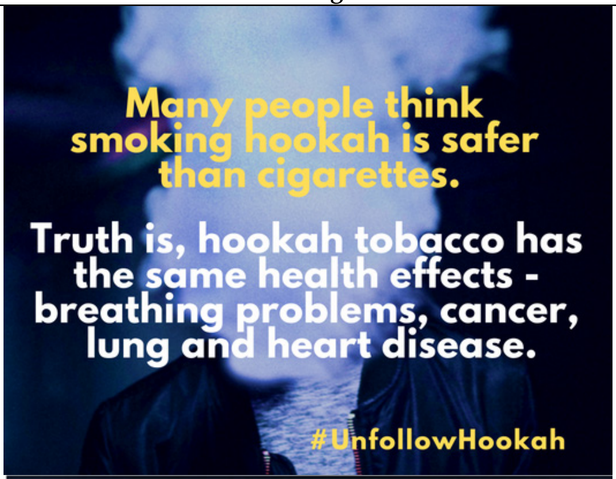  |
| H2:<br>Harms 2 | 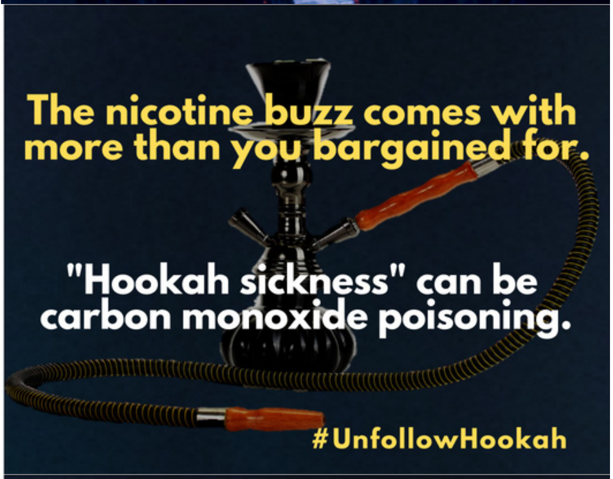  |
| HS1:<br>Harms/Social 1 | 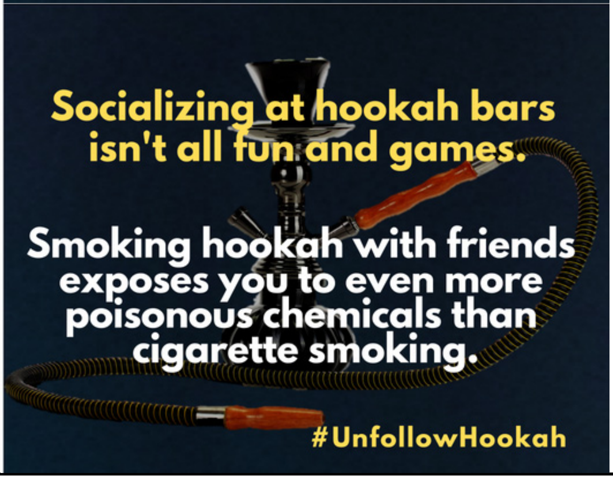  |

HS2:
Harms/Social 2

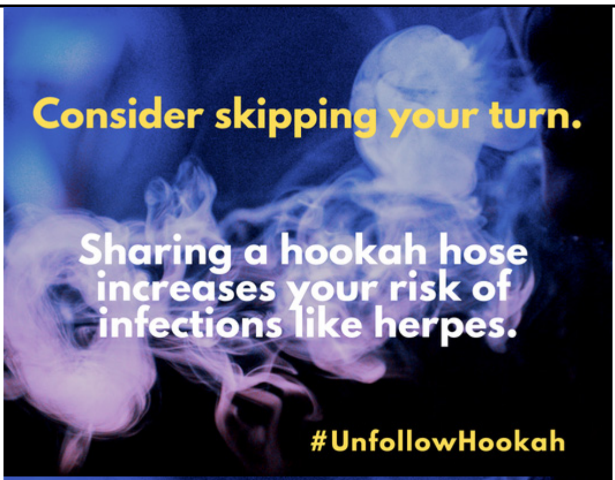


HF1:
Harms/Flavors 1

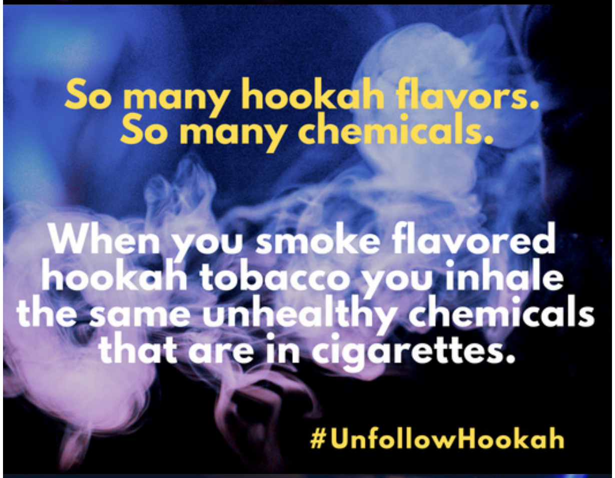


HF2:
Harms/Flavors 2

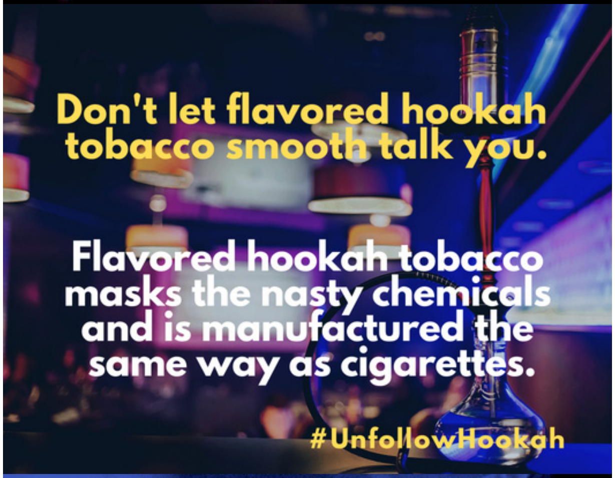


A1:
Addiction 1

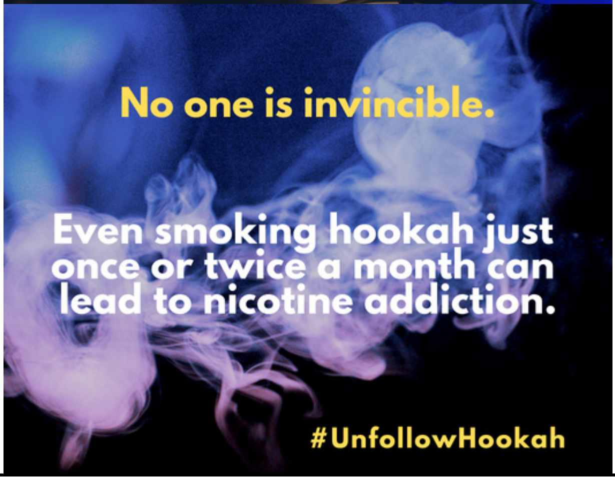

| | |
|---|---|
| A2: Addiction 2 | 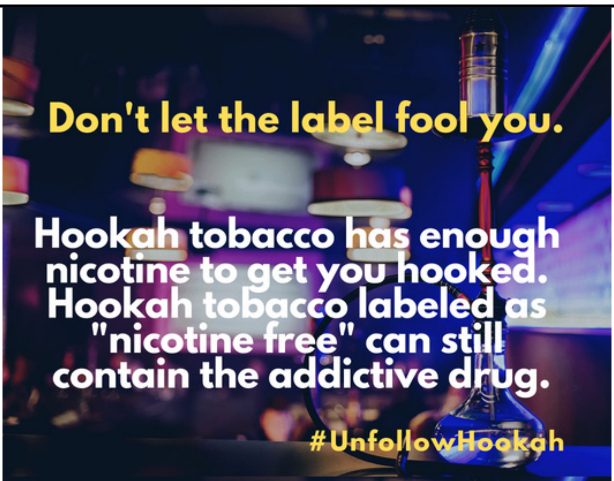  |
| AS1: Addiction/Social 1 | 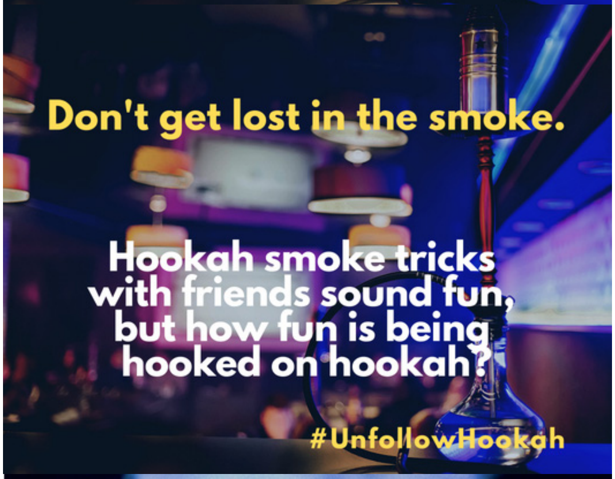  |
| AS2: Addiction/Social 2 | 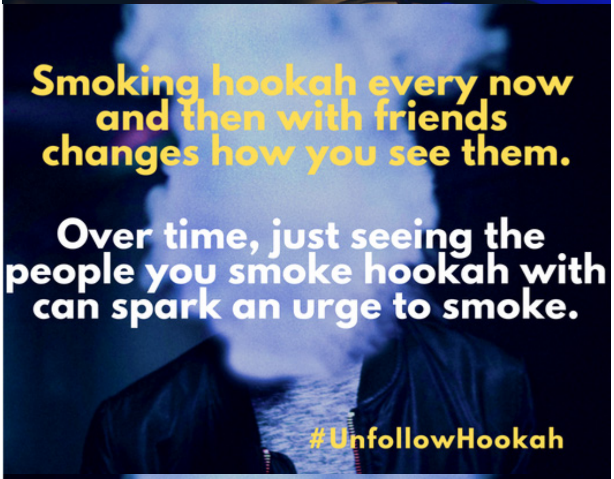  |
| AF1: Addiction/Flavors 1 | 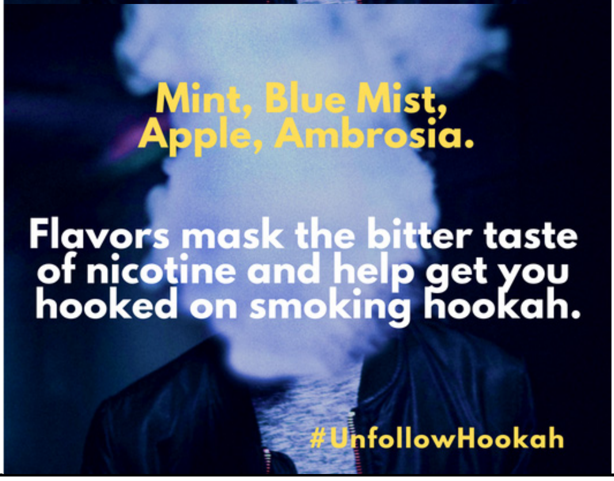  |

| | |
|---|---|
| AF2:<br>Addiction/Flavors 2 | 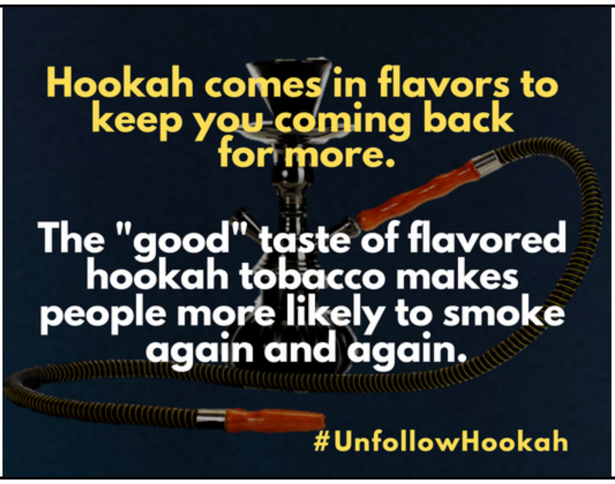 |
